# Supplementary material for: Effect of temperature on sporulation and spore development of giant kelp (Macrocystis pyrifera)
Source: PLoS One. 2022 Dec 8;17(12):e0278268. doi: 10.1371/journal.pone.0278268 (PMC9731429; doi:10.1371/journal.pone.0278268)
Supplement: Attachment — Submitted filename: PLOSReviewerComments.docx [file pone.0278268.s001.docx]

This manuscript examines the effects of temperature on giant kelp (M. pyrifera) in its earliest, microscopic life stages. The authors found a significant increase in spore release with increasing temperatures, but they also found the growth rate of germlings significantly decreased with increasing temperatures. Overall, this manuscript offers insight into which life stages may be vulnerable to rising ocean temperatures and what mechanisms may drive kelp loss in a changing ocean climate. This work will be particularly helpful for future conservation and restoration efforts for managers hoping to maintain kelp populations in heat-stressed regions.

My primary comments for the manuscript are the following:

- During revision, this manuscript should undergo a grammatical review. There are multiple instances of missing or misused punctuation. Other sentences are missing conjunctions (e.g., “and”) or articles (e.g., “the”). It does not detract from the overall clarity of the manuscript, but it is somewhat distracting.
- In the discussion section, the authors should add language to consider the potential effects of elevated temperature on subsequent life stages. If they are using their findings to advocate for the selection of thermally-tolerant individuals, there should be additional studies cited that support this approach, particularly in aquaculture settings, and they should aim to demonstrate that the effects of thermal stress will not be carried forward as the individual continues to grow.
- In the conclusion (or the discussion section), the authors should also spend more time placing their findings in a broader context of global kelp populations and the stress they face from increasing ocean temperatures.

Please see my additional line-by-line comments below:

Line 28 - The phrase in the abstract should read “spore settlement was the developmental phase most drastically effected by increasing temperature.”

Line 49-52 – The sentence beginning with “Temperature plays a pivotal role…” is a run-on sentence. I would suggest revising it to be two separate sentences.

Line 87 – Could you provide a bit more description about the site from which kelp was sampled? Depth/oceanographic seasons/temperature ranges/etc. Is this a region with abundant, constant, or flashy kelp populations?

Line 103 – Can you please add in a brief justification for the temperature range used in this experiment? Is this the current/projected temperature range for M. pyrifera populations in NZ? (Could re-use citations from Lines 64/65.)

Line 165 – I would also recommend adding in an explicit mention of the model structures. Did you begin with an interaction term for the fixed effects in every model? The interaction is often mentioned in the results, and appears in Table 1, so the interaction effect should first be mentioned in this paragraph if that was the default structure.

Line 168 – Where will this dataset be made available?

Figures 1 & 2 – The difference in spore production and settlement between the warmest treatment and the rest is quite striking!

For Figures 1-4, you might consider making it a single, four-paneled figure, so that readers could compare the outcomes at various stages in the life cycle across temperature treatments. I think they could certainly still be kept separate, but just a thought.

Line 213 – After a mention of the greatest increase in diameter, you should also mention the greater tube diameters measured on Day 1 for temperatures between 12 and 19C. These all appear noticeably larger than the 9 and 11C treatments, not to mention the warmest treatments (23-26C).

Table 1 – Why did you choose to keep Day and Temperature*Day effects in the Germination model when they do not appear to be significant? It seems the other models were trimmed to include only significant fixed effects, so if you have a study design or scientific basis for keeping non-significant effects in, I would add those justifications to the Germination results section.

Line 242 – Isn’t increased spore production also potentially a compensatory response of kelp to counteract the likelihood of increased mortality as a result of increased temperatures? Could you discuss/present examples of this kind of response in other algae/marine species?

Line 265 – If you propose using this strategy for selection of thermally tolerant individuals, you should also discuss the possibility of heat damaging cells prior to dropping temperature back to “normal” levels. If they are damaged during the initial, warmer period, additional research is necessary to determine if they are still viable for germination.

Line 271 – Similar to my comment above, if you advocate for choosing thermally-tolerant individuals, you must also better explain/justify how that tolerance may carry over into other life stages.

Line 287 – The information in the sentence beginning with “Temperature above 19.8C…” should be presented much earlier in the discussion so the reader can be reminded of the biologically relevant temperature range as they read through the remainder of the discussion.

Line 291 – I would like to see some more discussion of the conditions in southern New Zealand from where these kelp were sampled (see my comment for the Methods/Site section), and whether this area is subject to a wide range in temperatures or rapidly rising temperatures, buffered by upwelling, etc. More language describing how this population might relate to other global kelp populations would help to better place this study in a broader context.
